# Supplementary material for: Establishment and validation of systemic inflammatory index model and risk assessment of PVT in cirrhosis after splenectomy—a retrospective study
Source: PeerJ. 2025 May 12;13:e19254. doi: 10.7717/peerj.19254 (PMC12080476; doi:10.7717/peerj.19254)
Supplement: Supplemental Information 2 [file peerj-13-19254-s002.docx]

**Table S Description of abbreviations**

| **Abbreviations** | **Full title in English** |
| --- | --- |
| BMI | Body mass index |
| ASA | American Society of Anesthesiologists |
| Child-pugh | Child-pugh classification |
| HV | Viral hepatitis |
| SP | Surgical procedures |
| PVB | Platelet variability |
| WBC | White blood cell |
| NLR | Neutrophil to lymphocyte ratio |
| PLR | Platelet-to-lymphocyte ratio |
| dNLR | Derived granulocyte lymphocyte ratio |
| LMR | Lymphocyte monocyte ratio |
| CAR | C-reactive protein to albumin ratio |
| FAR | Fibrinogen to albumin ratio |
| PCT | Procalcitonin |
| Il-6 | Interleukin-6 |
| PT | Prothrombin time |
| INR | International normalized ratios |
| SPT | Spleen thickness |
| DPV | Diameter of Portal Vein |
| DSV | Diameter of the splenic vein |
| DII | D-II polymers |
| INR | International normalized ratio |
| FIB | Fibrinogen |
| ATIII | Antithrombin III |
| PS | Postoperative |
